# Development of a clinical risk score for pain and function following total knee arthroplasty: results from the TRIO study

**DOI:** 10.1093/rap/rky021

**Published:** 2018-05-29

**Authors:** Joanna Shim, David J Mclernon, David Hamilton, Hamish A Simpson, Marcus Beasley, Gary J Macfarlane

**Affiliations:** 1 Epidemiology Group, School of Medicine, Medical Sciences and Nutrition, University of Aberdeen; 2 Aberdeen Centre for Arthritis and Musculoskeletal Health, University of Aberdeen; 3 Medical Statistics Team, Institute of Applied Health Sciences, School of Medicine, Medical Sciences and Nutrition, University of Aberdeen, Aberdeen; 4 Department of Orthopaedics & Trauma, School of Clinical Sciences, University of Edinburgh, Edinburgh, UK

**Keywords:** knee pain, osteoarthritis, total knee arthroplasty, prediction modelling, clinical risk score, model calibration, model discrimination

## Abstract

**Objectives:**

The aim was to develop and validate a simple clinical prediction model, based on easily collected preoperative information, to identify patients at high risk of pain and functional disability 6 months after total knee arthroplasty (TKA).

**Methods:**

This was a multicentre cohort study of patients from nine centres across the UK, who were undergoing a primary TKA for OA. Information on sociodemographic, psychosocial, clinical and quality-of-life measures were collected at recruitment. The primary outcome measure for this analysis was the Oxford knee score (OKS), measured 6 months postoperatively by postal questionnaire. Multivariable logistic regression was used to develop the model. Model performance (discrimination and calibration) and internal validity were assessed, and a simple clinical risk score was developed.

**Results:**

Seven hundred and twenty-one participants (mean age 68.3 years; 53% female) provided data for the present analysis, and 14% had a poor outcome at 6 months. Key predictors were poor clinical status, widespread body pain, high expectation of postoperative pain and lack of active coping. The developed model based on these variables demonstrated good discrimination. At the optimal cut-off, the final model had a sensitivity of 83%, specificity of 61% and positive likelihood ratio of 2.11. Excellent agreement was found between observed and predicted outcomes, and there was no evidence of overfitting in the model.

**Conclusion:**

We have developed and validated a clinical prediction model that can be used to identify patients at high risk of a poor outcome after TKA. This clinical risk score may be an aid to shared decision-making between patient and clinician.

**Key messages**

- Predictors of poor outcome following total knee arthroplasty included illness attitudes and behaviours and clinical factors.
- A model based on easily measurable variables demonstrates good performance.
- The prediction tool developed can be an aid to shared decision-making between patient and clinician.

## Introduction

Total knee arthroplasty (TKA) is one of the most common and effective treatments for severe knee OA, with >100 000 knee replacements performed in the UK annually [1, 2]. Despite success in reduction of pain after knee replacements, ∼20–30% of patients continue to experience pain and limited function after their TKA, which cannot be explained entirely by biomedical factors [3–5].

Clinical determinants of outcomes after TKA that have been shown consistently to be related to outcome across reviews include preoperative pain and function, pain at other sites and aspects of surgery (longer duration of surgery, lengthy wait times) [6–12]. For other factors, the evidence is not consistent and may be related to the outcome studied. For example, one systematic review focusing on patients’ characteristics found that younger age and being male were related to risk of revision, older age was associated with increased risk of mortality and poorer function after TKA, but age and sex did not influence postoperative pain [13].

The importance of psychosocial and individual factors as predictors of musculoskeletal outcomes has also been increasingly recognized [14–16]. Adverse psychological factors, such as anxiety and depression, may have an effect on pain perception and mediate the development of chronic pain and disability [17, 18]. The relationship between psychosocial factors and TKA outcomes has been examined in several systematic reviews, which have consistently indicated poor preoperative mental health and pain catastrophizing to be strongly associated with greater postoperative pain and functional disability [7, 10, 19]. Limited or conflicting evidence was found for other psychological factors. It is clear from the reviews that there is a lack of consensus on the most important clinical and psychological risk factors for poor outcomes after TKA.

Although the decision to operate is primarily based on radiographic evidence of OA and the patient’s report of symptoms, variation in the use of surgery reflects the different beliefs among patients and surgeons regarding the risks and benefits of surgery. In a US-based study, Riddle *et al.* [20] reported that one-third of cases reviewed that underwent knee replacement surgery were ‘inappropriate’ and as a group, these patients demonstrated worse outcomes. The fact that surgery might not be successful for certain patients still highlights the need for robust predictive models to inform the clinical decision-making process.

Therefore, our study aimed firstly, to predict the impact of pain and functional disability 6 months after TKA using routinely collected patient preoperative information and secondly, to incorporate this information into a clinical prediction tool.

## Methods

The Targeted Rehabilitation to Improve Outcome—preoperative predictors of unfavourable outcome following knee arthroplasty study was a multicentre cohort study to investigate potential preoperative predictors of poor outcome after TKA. The study recruited from nine participating centres across the UK between December 2013 and July 2016. The study was conducted alongside a randomized controlled trial of targeted rehabilitation to improve outcome after TKA [21].

Adults aged ≥16 years, undergoing primary TKA for OA, were invited to take part in the study either by letter or in person at a clinic visit before surgery. Participants were excluded if they: were undergoing a revision TKA or fully constrained knee arthroplasty; had a TKA for a diagnosis other than OA; or had existing medical conditions, such as stroke, or other musculoskeletal conditions that cause a limitation of function. Participants completed a questionnaire at the time of recruitment, and consent was obtained for access to medical records for research purposes. Follow-up questionnaires were posted to participants 6 weeks, 3 and 6 months after surgery. Ethical approval was granted by the office for Research Ethics Committees Northern Ireland (ORECNI) (13/NI/0101).

### Preoperative questionnaire

The preoperative questionnaire included the following items.

#### Sociodemographic factors

Age, sex, marital status, socioeconomic status (highest education level achieved) and employment status were measured.

#### Clinical factors

Clinical factors measured included duration of knee pain, baseline pain and function using the Oxford knee score (OKS) [22] and the chronic pain grade (CPG) [23]. The CPG contains seven items that allow respondents to be classified into five categories: grade 0 (no pain), grade I (low disability/low intensity), grade II (low disability/high intensity), grade III (high disability/moderately limiting intensity) and grade IV (high disability and highly limiting disability). Body manikins were used to determine whether participants met the definition of chronic widespread pain used in the ACR criteria for FM [24].

The sleep problem scale consists of four questions, rated on a six-point frequency rating scale, ranging from zero (not at all) to five (22–31 days/month) [25]. Sleep disturbance was defined as a mean score ≥4, corresponding to at least 15 troubled nights per month [25]. Self-reported co-morbidities in this cohort were also recorded.

#### Psychosocial factors

The illness attitude scales [26, 27] measure personal attitudes, fears and beliefs associated with hypochondriasis and abnormal illness behaviour. It consists of nine subscales, each with three items on a 0–4 Likert scale. Scores are summed to give the total illness attitude scales score, with a higher score representing greater hypochondriacal fears and beliefs.

Among participants who reported that they had aches or pains lasting 1 day or longer in the past month, the Vanderbilt pain management inventory was used to assess chronic pain coping strategies [28]. This questionnaire consists of 18 items, rated on a five-point frequency Likert scale. From these data, two subscales can be calculated; active coping score and passive coping score. High scores indicate a high use of active and passive coping strategies, respectively.

Patient expectations of pain, and limitations in everyday activities after TKA were measured using visual analog scales; 0 representing not at all painful or not limited at all, and 100 very painful or greatly limited, respectively [29].

#### Mental and physical health

Mental and physical health was measured by the hospital anxiety and depression scale (HADS) [30] and the patient-reported outcomes measurement information system 10 (PROMIS-10) global health questionnaire [31]. The HADS is a 14-item questionnaire, with seven items measuring anxiety and seven items measuring depression. Each item is rated on a 0–3 Likert scale, with higher scores indicating poorer mental health. The PROMIS-10 questionnaire has 10 items that allow the global physical health and global mental health sub-scales to be derived. Scores range from 4 to 20, with higher scores indicating better health.

#### Quality of life

The EuroQoL-5 dimension (EQ-5D) is a measure of quality of life [32]. It consists of five dimensions: mobility, self-care, usual activities, pain/discomfort and anxiety/depression, rated on a three-point scale. Each EQ-5D profile was converted to a single summary index based on the valuation of health states in the UK. A score of 1.0 indicates the best possible health.

The outcome for this analysis was the OKS [33], measured 6 months postoperatively by postal questionnaire. The OKS measures the impact of pain and functional disability in patients undergoing knee replacement [34, 35]. Poor outcome was defined by a score of ≤26 (out of a maximal score of 48) according to the modified Kalairajah classification [36].

### Statistical analysis

The study aimed to recruit 750 participants, and if 80% of participants (*n* = 600) provided follow-up data, this would give 80% power to detect an odds ratio of 1.5 for a poor outcome, comparing the highest tertile with the other two tertiles of exposure. Descriptive statistics were carried out to describe the study sample, and the normality of individual variables was assessed. Categorical variables, the sleep problem scale, the CPG and the HADS anxiety and depression were categorized according to standard cut-offs.

In preparation for the modelling, the relationship between continuous predictor variables and the observed logarithmic odds of a poor outcome were assessed for linearity. Health scores measured by the EQ-5D and the PROMIS-10 questionnaire, measures of active and passive coping strategies determined by the Vanderbilt pain management inventory, patient expectations of outcomes after surgery and illness attitude scores were analysed as continuous variables. However, a maximal health index of one in the EQ-5D results in regression coefficients (expressed as change in outcome per one unit increase in predictor) that are not intuitive to interpret, and values were therefore multiplied by 10 for the purpose of the univariable and multivariable analyses. Logistic regression analysis was used to explore the association between each of the potential preoperative predictor variables and the OKS at 6 months. In the univariable analysis, variables showing an association with a significance level of *P* < 0.2 were candidates for entry into a forward stepwise regression as part of a bootstrap selection process, as described below. Entry and removal criteria for the stepwise models were *P* ≤ 0.1 and *P* > 0.15, respectively. We used stepwise regression to suggest predictor variables for the model, followed by the incorporation of clinical knowledge. Associations were expressed as odds ratios (ORs) with 95% CIs. To aid clinical decision-making, a simplified point-based risk-scoring system was developed using coefficients from the final model [37].

Multiple imputation with chained equations was used to impute missing predictor data with the aim of reducing bias and improving efficiency; 20 imputed data sets were generated [38, 39]. Detailed descriptions of the post-estimation procedure can be found in supplementary Appendix S1, available at *Rheumatology Advances in Practice* online.

Model discrimination was quantified using the area under the receiver operating characteristic curve or concordance (*c*) statistic to estimate predictive accuracy. A *c*-statistic value of one represents perfect discrimination, and a *c*-statistic of 0.5 indicates a discriminative value equivalent to chance [40]. A pooled *c-*statistic of the 20 imputed data sets was calculated. A shrinkage estimate was also calculated to assess overfitting. A shrinkage estimate of <0.8 would reflect a need for shrinkage of the regression coefficients in a prediction model using methods such as lasso or ridge regression [41].

Model calibration, which refers to the agreement between the observed and predicted probabilities, was also assessed using calibration-in-the-large [42]. This indicates whether the predictions are systematically too low or too high.

Overfitting occurs when a model is too strongly tailored to the specifics of the sample population used in development such that it predicts well for patients within the derivative cohort but is not generalizable to other samples [41]. A bootstrap resampling technique was used to test for overfitting. Details of the bootstrap approach can be found in supplementary Appendix S1, available at *Rheumatology Advances in Practice* online. Data were analysed using STATA version 14.0 (Stata Corp, College Station, TX) and Rstudio version 1.0.143 (RStudio Inc., Boston, MA).

## Results

Seven hundred and twenty-one of the 972 (75.7%) participants completed and returned the baseline and 6-month follow-up questionnaires and were eligible for this analysis. The mean age of the participants was 68.6 years, there was an even gender split, and approximately half were educated to secondary-school level (Table 1). Most participants were retired (56.5%), but approximately one in four were still working either full time or part time. Ninety-nine patients (14.1%) met the definition of poor outcome at 6 months post-TKA.

**Table 1:** Characteristics of the study population

Predictor		No. of respondents
Demographic and socioeconomic characteristics		
Age, median (IQR), years	68.6 (63.3–74.6)	721
Female, *n* (%)	379 (52.6)	721
Marital status, *n* (%)		719
Single	35 (4.9)	
Married	485 (67.5)	
Widowed	100 (13.9)	
Divorced	67 (9.3)	
Separated	8 (1.1)
Co-habiting	24 (3.4)	
Education, *n* (%)		719
Secondary school	356 (49.5)
Apprenticeship	81 (11.3)
Further education college	188 (26.2)
University degree	69 (9.6)
Further degree	25 (3.5)
Centres		
Edinburgh	242 (33.6)	721
Aberdeen	118 (16.4)	
Royal Orthopaedic Hospital	146 (20.3)	
Weston General Hospital	45 (6.2)	
Barts Health NHS Trust	17 (2.4)	
Warrington	20 (2.8)	
Fife	67 (9.3)	
Dudley	13 (1.8)	
Pennine Acute	53 (7.4)	
Work		
Current employment status, *n* (%)		703
Working full time	117 (16.6)	
Working part time	68 (9.7)	
Retired	397 (56.5)	
Unable to work because of illness or disability	41 (5.8)	
Student	0	
Unemployed and looking for work	6 (0.9)	
Not looking for paid employment	74 (10.5)	
Clinical factors		
Duration of knee pain, median (IQR), years	7.2 (2.0–10.0)	699
Baseline Oxford knee score, mean (IQR)	20.6 (15.0–26.0)	709
Chronic pain grade, *n* (%)		664
No pain, grade 0	126 (19.0)	
Low disability and low intensity, grade I	55 (8.3)	
Low disability and high intensity, grade II	175 (26.4)	
High disability and moderate intensity, grade III	145 (21.8)	
High disability and high intensity, grade IV	163 (24.6)	

IQR: interquartile range.

### Univariable analysis

There were several preoperative factors that predicted a poor outcome (see Table 2). Firstly, clinical status: severe chronic pain (CPG grade IV; OR = 11.25, 95% CI: 3.92, 32.30), chronic widespread pain (OR = 2.34, 95% CI: 1.30, 4.19), and a high number of co-morbidities (≥4 co-morbidities: 3.75, 95% CI: 1.90, 7.40). In contrast, a better OKS was associated with reduced risk of poor outcome (0.87/unit increase in score; 0.84–0.91). Secondly, psychosocial factors: illness attitudes were strongly related to poor outcome; for every one point increase in illness attitude score (OR = 1.03, 95% CI: 1.01, 1.05), the risk of poor outcome increased. Among participants who had reported aches or pains, the odds of a poor outcome also increased for every unit increase in passive coping score (OR = 1.08, 95% CI: 1.05, 1.12), whereas poor outcome was less likely for every unit increase in active coping strategies (OR: 0.87, 95% CI: 0.83, 0.92). Expectations were strongly associated with poor outcome; for every one point increase in expected knee pain after recovery (OR = 1.01, 95% CI: 1.01, 1.02) or expected limitations in everyday activities (OR = 1.02, 95% CI: 1.01, 1.02), the risk of poor outcome increased. Thirdly, mental health: severe anxiety (OR = 2.58, 95% CI: 1.48, 4.49) and depression (OR = 3.67, 95% CI: 1.88, 7.15) were associated with poor outcome, and for every one unit increase in the PROMIS mental score, the risk of poor outcome decreased (OR = 0.93, 95% CI: 0.89, 0.97). Finally, poor outcome was less likely among those with good preoperative physical health (PROMIS-physical health) and quality of life (EQ-5D).

**Table 2:** Univariable associations between individual preoperative variables and poor outcome

Predictors	Persons with poor outcomea	Total, n	Odds ratio (95% CI)
Sociodemographic factors			
Age, years	67.8 (9.0)	704	0.99 (0.96, 1.01)
Sex			
Female	55 (15.1)	704	Reference category	
Male	44 (12.9)		0.84 (0.54, 1.28)
Clinical factors			
Duration of knee pain, years	8.4 (7.6)	682	1.02 (0.998, 1.05*)
Baseline Oxford knee score; per unit (0–48)	15.2 (6.8)	695	0.87 (0.84, 0.91*)
Chronic pain grade			
No pain, grade 0	4 (3.3)	651	Reference category	
Low disability and low intensity, grade I	2 (3.8)		1.16 (0.21, 6.53)
Low disability and high intensity, grade II	12 (7.1)		2.25 (0.71, 7.17)
High disability and moderate intensity, grade III	24 (16.7)		5.90 (1.98, 17.54*)
High disability and high intensity, grade IV	45 (27.6)		11.25 (3.92, 32.30*)
Chronic widespread pain			
No	78 (12.5)	697	Reference category	
Yes	18 (25.0)		2.34 (1.30, 4.19*)
Sleep problem scale			
Mildly sleep disturbed (≤15 nights)	77 (13.0)	699	Reference category	
Severely sleep disturbed (>15 nights)	21 (19.8)		1.66 (0.97, 2.83*)
Co-morbidities			
≤1 co-morbidities	14 (8.5)	704	Reference category	
2–3 co-morbidities	53 (12.8)		1.58 (0.85, 2.93)
≥4 co-morbidities	32 (25.8)		3.75 (1.90, 7.40*)
Psychosocial factors			
Illness attitude score; per unit (0–108)	31.9 (13.4)	655	1.03 (1.01, 1.05*)
Active coping; per unit (7–35)	21.1 (4.6)	562	0.87 (0.83, 0.92*)
Passive coping; per unit (11–55)	33.6 (7.6)	547	1.08 (1.05, 1.12*)
Expectations of pain after recovery; per unit (0–100)	51.4 (29.0)	685	1.01 (1.01, 1.02*)
Expectations of limitations after recovery; per unit (0–100)	43.5 (25.4)	685	1.02 (1.01, 1.02*)
Mental and physical health			
HADS^b^ anxiety			
Mild to moderate anxiety	84 (12.8)	702	Reference category	
Severe anxiety	15 (34.9)		2.58 (1.48, 4.49*)
HADS depression			
Mild to moderate depression	78 (12.5)	702	Reference category	
Severe depression	21 (26.9)		3.67 (1.88, 7.15*)
PROMIS^c^ mental health; per unit (4–20)	42.6 (5.6)	696	0.93 (0.89, 0.97*)
PROMIS^c^ physical health; per unit (4–20)	35.0 (3.6)	691	0.82 (0.77, 0.87*)
Quality of life			
EQ-5D; per 10th of a unit (−0.5, 1.0)	2.6 (1.8)	685	0.74 (0.65, 0.83*)

^a^For categorical variables, the number and percentages of persons with poor outcome are reported. Means (s.d.) of persons with poor outcome are reported for continuous variables.

^b^Hospital anxiety and depression scale.

^c^Patient-reported outcomes measurement information system.

* *P* < 0.2.

There were other factors that were not significantly associated with outcome but met the criteria for being considered in the multivariable model: severely disturbed sleep and a long duration of knee pain. In contrast, age and gender were not related to outcome and were not considered further.

### Model development and validation

Of the factors eligible for inclusion in the multivariable models (*P* < 0.2), four were entered and retained in the final model predicting poor outcome: low preoperative OKS, chronic widespread pain, high expectations of knee pain after recovery and lack of active coping strategies (Table 3). The model demonstrated good discrimination between patients at high and low risk of poor outcome after TKA, as indicated by a pooled *c-*statistic of 0.78 (pooled estimates of the 20 imputations). The final predictive model had a sensitivity of 82.8%, a specificity of 60.7% and a positive likelihood ratio (LR) of 2.11 at the optimal cut-off identified by Youden’s index (*J*).

**Table 3:** Predictors of poor outcome in a multivariable stepwise regression model

Predictors	Adjusted odds ratio (95% CI)
Oxford knee score (per unit increase in score)	0.89 (0.86, 0.93)
Expectations of knee pain after recovery (per unit increase in score)	1.01 (1.005, 1.02)
Active coping (per unit increase in score)	0.91 (0.86, 0.96)
Chronic widespread pain	1.65 (0.86, 3.17)

Excellent agreement was found between observed and predicted probabilities. The estimate obtained with the bootstrap resampling was very close to the original estimate across the 20 imputed data sets. After correcting for optimism, the average *c*-statistic was 0.77. This suggested a reliable optimism-corrected *c*-statistic. Calibration-in-the-large showed no evidence of systematic overestimation or underestimation of the predicted probability of outcome. The average calibration-in-the-large was 0.16 (95% CI: −0.07, 0.34), which indicated that there was no evidence of overfitting in the model.

### Clinical prediction tool

**Fig. 1:**
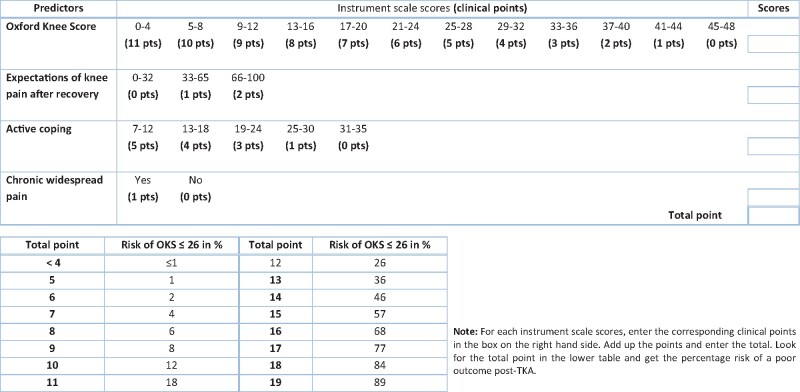
Points-based risk-scoring system for estimation of poor outcome [defined as Oxford knee score (OKS) ≤ 26] after total knee arthroplasty Scores range from 0 to 19 points.

A simple risk-scoring system was developed from the multivariable model, which can be found in supplementary Appendix S2, available at *Rheumatology Advances in Practice* online. Scores range from 0 to 19, with higher scores corresponding to higher risk of poor outcome at 6 months post-TKA. Risk estimates are attached to each point total, as shown in Fig. 1. Two case studies demonstrating the relationship between the estimated risks of the prediction tool and those from the logistic regression model are available in supplementary Appendix S3, available at *Rheumatology Advances in Practice* online.

## Discussion

Expectations (of poor outcome) and behaviour (lack of active coping) in addition to clinical factors (poor preoperative knee status and chronic widespread pain) were key predictors of a poor outcome in persons undergoing TKA. A clinical prediction model based on these factors demonstrated good performance in identifying patients who had poor outcome based on OKS.

A strength of our study is the multicentre nature and large sample size. We have measured a range of patient-reported factors, focusing in particular on those that have been shown to predict outcome for musculoskeletal disorders, and specifically, pain. Robust statistical methods, such as multiple imputation and bootstrap resampling, were used to strengthen the development of this clinical prediction tool. Multiple imputation encourages statistical efficiency, especially when missing data are assumed to be missing at random, which is plausible in the context of this study [43]. With many variables and rare events, there is a risk of overfitting the model. To test for this, we measured the shrinkage factor, an indicator for reliable estimations, to determine whether there was a need to reduce the regression coefficients using a shrinkage method (e.g. lasso), and overfitting was not indicated (shrinkage factor > 0.8) [41].

Limitations of our study include the fact that only a few clinical factors were measured and some, such as joint damage or BMI, were not available. Although BMI is often associated with many conditions, including OA, there is no evidence in the literature to suggest that BMI is a clinically important predictor of postoperative outcome [44, 45]. Although the absolute risk remains small, higher BMI is, however, associated with an increased relative risk of revisions and post-surgical complications, which are important factors to consider in decision-making [46–48]. There were also no intra-operative factors collected, some of which have been related to poor outcome. However, as the purpose was to develop a clinical prediction tool to aid shared decision-making by the clinician and the patient about proceeding to knee replacement surgery, then by *de facto* this must be based only on factors available at this time. At the optimal cut-off for clinical use, there was a sensitivity and specificity of 82.8 and 60.7%, respectively, with a positive LR of 2.11. Although the LR of the positive test falls below the recommended value for a strong diagnostic test (LR = 5), it is comparable to other prediction rules reported in the literature (e.g. Lungu *et al.* [49]). Our study predicted a binary outcome, using a recommended cut-off of the OKS. We tested our model using other cut-offs that have been proposed (OKS ≤19/>19) [50] and also developed a model that predicted the score rather than a binary state. Each of these alternative strategies produced very similar predictive models (data not shown).

To our knowledge, only two other studies have translated determinants of TKA outcomes into a clinical prediction rule [44, 49]. Lungu *et al.* [49] explored an extensive list of potential predictors and included 5 of the 24 items from the WOMAC questionnaire in their prediction rule. Four of the questions were specific to preoperative function and the other measured stiffness. Their model, based on a small sample size of 141 patients, demonstrated good overall predictive validity for outcomes 6 months postsurgery: sensitivity 82%, specificity 72% and positive LR of 2.9. The second study was an extensive programme of work funded by the National Institute for Health Research [44]. Using data from the Knee Arthroplasty Trial, Arden *et al.* [44] developed the Clinical Outcomes in Arthroplasty Study knee model to predict 12-month postoperative OKS. This model included patient characteristics (age, sex, preoperative OKS, BMI, deprivation score, SF-12 mental component summary score) and clinical factors [the American Society of Anesthesiologists grade (a measure of fitness for surgery), co-morbidities, previous knee surgery, fixed flexion deformity, valgus or varus deformity and preoperative anterior cruciate ligament state] [44]. Internal validation of the model demonstrated overall good discrimination (*R*^2^ = 20%) and calibration, but it did not perform well in their validation cohort [44]. They attributed this to fundamental differences in patient characteristics, surgical techniques and implants, the proportion of missing data and varying proxy variables between the development and validation cohorts. A further cost-utility analysis did not find the Clinical Outcomes in Arthroplasty Study knee model to be cost-effective; therefore, the implementation in practice could not be recommended. It is of note that previous models are solely focused on clinical factors, whereas the evidence from this study and others [7, 10, 19] demonstrates that outcome is influenced by both clinical factors and psychosocial factors (including patient beliefs and health behaviour). It is likely that any clinical prediction model will need to incorporate both these domains to be optimal in predicting outcomes.

Our findings highlight the importance of biopsychosocial assessment in patients undergoing TKA. Alattas *et al.* [50], in a systematic review that included 10 studies, found consistent evidence for the role of anxiety and some evidence for the role of depression in predicting poor outcome. We found that people with high expectations of knee pain after recovery also have poorer outcome. Taking into account their condition and their requirements, patients may make a realistic assessment of their outcome. However, pessimism has been linked to long-term poorer physical health, even when controlling for the health status at the time of pessimism [51]. Misplaced adverse beliefs may influence one’s perception of events and affect the way we cope [16]. Studies have found that active coping strategies, such as remaining active and positive refocusing, are associated with less pain and functional impairment [28, 52], whereas adopting passive coping strategies, such as catastrophizing, has been related to poorer functional outcomes [16]. The role of psychosocial factors in predicting outcome is important because such factors are potentially modifiable preoperatively and if the relationship is causal, could improve outcome. Cognitive and other behavioural therapies, which can include focusing on behavioural activation, pacing and changes in lifestyle, can alter patients’ expectation and coping style, and indeed, have been shown to have positive effects on pain experience and positive coping measures [53].

The purpose of designing a clinical prediction tool is not to determine who should and should not undergo TKA but instead to act as an aid to shared decision-making between the patient and clinician in terms of highlighting patients at higher risk of a poor outcome and also establishing realistic expectations of postoperative pain and function.

In conclusion, we have developed a prediction model for outcome after TKA, including both clinical factors and patient attitudes and behaviour in terms of self-management. Future work may investigate the validation of the model in another cohort and its impact on clinical decision-making. The results also offer the possibility that modifying illness beliefs and behaviours may result in better TKA outcomes.

## Supplementary Material

rky021_Supplementary_Data
