# Supplementary material for: Development of a clinical risk score for pain and function following total knee arthroplasty: results from the TRIO study
Source: Rheumatol Adv Pract. 2018 May 29;2(2):rky021. doi: 10.1093/rap/rky021 (PMC6251482; doi:10.1093/rap/rky021)
Supplement: rky021_Supplementary_Data [file rky021_supplementary_data.docx]

# Supplementary Data

# Appendix 1

**Post-estimation with multiple imputation**

Multiple imputation with chained equations (MICE) was used to impute missing predictor data. Specification of the imputation model was performed according to the recommendations described by van Buuren et al. (1999) and White et al. (2011); 20 imputed datasets were generated. In each of the imputed datasets, a model was fitted using STATA’s ‘swboot’ syntax to perform 200 bootstrapped repeats of forward stepwise logistic regression on potential predictor variables (Garrett 2000). The inclusion frequency or the number of times each variable entered the model from the 4000 bootstrapped procedures was calculated. The selection of variables for the final model was estimated based on predictors whose average inclusion frequency exceeded a threshold of 50% across the 20 imputed datasets (Garrett 2000). This process investigated the strength of the evidence that each variable is an independent predictor (Austin et al. 2004). After the independent predictors were determined from the bootstrap procedure, the logistic regression model with these predictors was applied to each of the 20 imputed datasets. The resulting coefficients from each of the 20 models were pooled into a combined estimate using Rubin’s rules (Rubin 1987).

**Internal validation of predictive logistic regression model using bootstrap resampling technique**

Internal validity was estimated with bootstrap resampling, which provides stable estimates with low bias (Steyerberg 2009). Bootstrapping was used to estimate the optimism of the prediction model; the decrease between performance in the bootstrap sample and the performance in the original sample. The following steps were carried out (Steyerberg 2009):

1. A model was constructed in the original sample. This model was selected using forward stepwise regression.
2. Using a bootstrap resampling method, 300 samples were randomly drawn from the original sample used to construct the model.
3. Forward stepwise regression was then applied to each bootstrapped sample and the *c*-statistic was calculated for each of the 300 models derived from the 300 samples (C_b_). Each of the 300 models were also applied to the original dataset and the *c*-statistic calculated for each (C_a_).
4. The difference between the two *c*-statistics for the bootstrapped sample and the original dataset was calculated (C_b_ – C_a_), and the average was obtained.

Process (1) to (3) was repeated for each of the 20 imputed datasets. The averaged value of (C_b_ – C_a_) was subtracted from the original *c-*statistic for each imputed dataset to provide the optimism‑corrected *c-*statistic, which is an estimate of internal validity.

# Appendix 2

**Table A. Scoring system to calculate the points associated with each of the categories of risk factors. Points were rounded to the nearest integer.**

| Predictors | β | Categories | Reference value (W) | β*(W-W_ref_) | Points= β*(W-W_ref_)  *B^[[1]](#footnote-1)^* | Rounded Points |
| --- | --- | --- | --- | --- | --- | --- |
| Oxford knee score | -0.112 | 45-48* | 46.5 (W_ref_) | 0 | 0 | 0 |
|  |  | 41-44 | 42.5 | 0.45 | 1 | 1 |
|  |  | 37-40 | 38.5 | 0.90 | 2 | 2 |
|  |  | 33-36 | 34.5 | 1.35 | 3 | 3 |
|  |  | 29-32 | 30.5 | 1.79 | 4 | 4 |
|  |  | 25-28 | 26.5 | 2.24 | 5 | 5 |
|  |  | 21-24 | 22.5 | 2.69 | 6 | 6 |
|  |  | 17-20 | 18.5 | 3.14 | 7 | 7 |
|  |  | 13-16 | 14.5 | 3.59 | 8 | 8 |
|  |  | 9-12 | 10.5 | 4.04 | 9 | 9 |
|  |  | 5-8 | 6.5 | 4.48 | 10 | 10 |
|  |  | 0-4 | 2 | 4.99 | 11.13 | 11 |
| Expectations of knee pain after recovery | 0.013 | 0-32* | 16 (W_ref_) | 0 | 0 | 0 |
|  |  | 33-65 | 49 | 0.44 | 0.98 | 1 |
|  |  | 66-100 | 83 | 0.89 | 1.98 | 2 |
| Active coping | -0.096 | 31-35* | 33 (W_ref_) | 0 | 0 | 0 |
|  |  | 25-30 | 27.5 | 0.53 | 1.18 | 1 |
|  |  | 19-24 | 21.5 | 1.11 | 2.47 | 2 |
|  |  | 13-18 | 15.5 | 1.69 | 3.76 | 4 |
|  |  | 7-12 | 9.5 | 2.26 | 5.05 | 5 |
| Chronic Widespread Pain | 0.502 | No* | 0 (W_ref)_ | 0 | 0 | 0 |
|  |  | Yes | 1 | 0.50 | 1.12 | 1 |

* Reference category

# Appendix 3

Table B presents two hypothetical case studies used to illustrate the clinical prediction tool (and correspondence with logistic regression model). In case 1, the predicted probability of a poor outcome for a patient with a good baseline OKS (40 out of 48), low expectations of knee pain after recovery (VAS 30 out of 100) with some active coping strategies (25 out of 35), and no chronic widespread pain is estimated to be less than 1.0%. Employing the model directly gives a risk of 0.9%. The patient in case study 2 presents with poor baseline OKS (13 scores), chronic widespread pain, high expectations of knee pain (70 out of 100) and minimal active coping strategies (8 out of 35) gives a risk estimate of poor outcome of 67.6%. This is comparable to the 71.7% produced by the logistic regression model.

**Table B. Examples of clinical case studies**

|  | Case Study 1 | | Case Study 2 | |
| --- | --- | --- | --- | --- |
| Clinical Prediction Model Items | **Value** | **Points** | **Value** | **Points** |
| Baseline OKS (score 0-48) | 40 | 2 | 13 | 8 |
| Expectations of knee pain (VAS 0-100) | 30 | 0 | 70 | 2 |
| Active coping score (score 7-35) | 25 | 1 | 8 | 5 |
| Chronic widespread pain (Yes/No) | No | 0 | Yes | 1 |
|  | **Total clinical point** | 3 |  | 16 |
|  | **Estimate of risk based on scoring system** | <0.010^[[2]](#footnote-2)^ |  | 0.676^[[3]](#footnote-3)^ |

OKS- Oxford Knee Score

1. Base constant (*constant B*) reflect the increase in risk associated with a 4-point increase in OKS (minimal clinically important difference is between 3 and 5 points); *B* = 4*β [↑](#footnote-ref-1)
2. The risk estimate based on the logistic model = 1.742 + (-0.112*40) + (0.013*30) + (-0.096*25) + (0.502*0) = - 4.75

   Risk estimate= 1 = 0.009

   1 + exp (-1 * - 4.75) [↑](#footnote-ref-2)
3. The risk estimate based on the logistic model = 1.742 + (-0.112*13) + (0.013*70) + (-0.096*8) + (0.502*1) = 0.93

   Risk estimate= 1 = 0.717

   1 + exp (-1 * 0.94) [↑](#footnote-ref-3)
